# Aortic valve stenotic area calculation from phase contrast cardiovascular magnetic resonance: the importance of short echo time

**DOI:** 10.1186/1532-429X-11-49

**Published:** 2009-11-19

**Authors:** Kieran R O'Brien, Ruvin S Gabriel, Andreas Greiser, Brett R Cowan, Alistair A Young, Andrew J Kerr

**Affiliations:** 1 Auckland Bioengineering Institute, University of Auckland, Auckland, New Zealand; 2 Cardiology Department, Middlemore Hospital and University of Auckland, Auckland, New Zealand; 3 Siemens AG Healthcare Sector, Erlangen, Germany; 4 Centre for Advanced MRI, University of Auckland, Auckland, New Zealand; 5 Department of Anatomy and Radiology, University of Auckland, Auckland, New Zealand

## Abstract

**Background:**

Cardiovascular magnetic resonance (CMR) can potentially quantify aortic valve area (AVA) in aortic stenosis (AS) using a single-slice phase contrast (PC) acquisition at valve level: AVA = aortic flow/aortic velocity-time integral (VTI). However, CMR has been shown to underestimate aortic flow in turbulent high velocity jets, due to intra-voxel dephasing. This study investigated the effect of decreasing intra-voxel dephasing by reducing the echo time (TE) on AVA estimates in patients with AS.

**Method:**

15 patients with moderate or severe AS, were studied with three different TEs (2.8 ms/2.0 ms/1.5 ms), in the main pulmonary artery (MPA), left ventricular outflow tract (LVOT) and 0 cm/1 cm/2.5 cm above the aortic valve (AoV). PC estimates of stroke volume (SV) were compared with CMR left ventricular SV measurements and PC peak velocity, VTI and AVA were compared with Doppler echocardiography. CMR estimates of AVA obtained by direct planimetry from cine acquisitions were also compared with the echoAVA.

**Results:**

With a TE of 2.8 ms, the mean PC SV was similar to the ventricular SV at the MPA, LVOT and AoV_0 cm _(by Bland-Altman analysis bias ± 1.96 SD, 1.3 ± 20.2 mL/-6.8 ± 21.9 mL/6.5 ± 50.7 mL respectively), but was significantly lower at AoV_1 _and AoV_2.5 _(-29.3 ± 31.2 mL/-21.1 ± 35.7 mL). PC peak velocity and VTI underestimated Doppler echo estimates by approximately 10% with only moderate agreement. Shortening the TE from 2.8 to 1.5 msec improved the agreement between ventricular SV and PC SV at AoV_0 cm _(6.5 ± 50.7 mL vs 1.5 ± 37.9 mL respectively) but did not satisfactorily improve the PC SV estimate at AoV_1 cm _and AoV_2.5 cm_. Agreement of CMR AVA with echoAVA was improved at TE 1.5 ms (0.00 ± 0.39 cm^2^) versus TE 2.8 (0.11 ± 0.81 cm^2^). The CMR method which agreed best with echoAVA was direct planimetry (-0.03 cm^2 ^± 0.24 cm^2^).

**Conclusion:**

Agreement of CMR AVA at the aortic valve level with echo AVA improves with a reduced TE of 1.5 ms. However, flow measurements in the aorta (AoV 1 and 2.5) are underestimated and 95% limits of agreement remain large. Further improvements or novel, more robust techniques are needed in the CMR PC technique in the assessment of AS severity in patients with moderate to severe aortic stenosis.

## Background

Accurate assessment of lesion severity is central to surgical decision making for patients with aortic stenosis. Transthoracic echocardiographic assessment is typically used to make this assessment; however, poor image quality due to limited acoustic windows and the experience of the operator may have a larger impact on reliable measurements than with other modalities [1]. For example poor jet alignment may cause the underestimation of jet velocities [2], the echo continuity equations assumes that the left ventricular outflow tract (LVOT) is circular when it is ovoid and the tracing of the Doppler velocity envelope and estimation of the LVOT's diameter are dependent on the analyst's experience. Currently, when transthoracic echo results are equivocal an invasive investigation such as cardiac catheterisation or transoesophageal echo may be required to estimate aortic valve area (AVA). Cardiovascular magnetic resonance (CMR) may be a useful alternative non-invasive modality.

Because CMR can measure flow volume and velocity, it is theoretically possible to estimate AVA using the continuity equation [3] or more directly from the flow volume and velocity-time integral (VTI) [4] sampled from a single acquisition at the valve level. To be accurate, the velocity and flow data obtained in these turbulent, high velocity jets must be accurate. Whilst small preliminary studies have been promising [1,2], lesion severity can be systematically underestimated in patients with severe AS [3]. Furthermore, in one of the largest cohort of AS patients studied with CMR to date, aortic flow sampled just above the stenotic aortic valve (AoV) systematically underestimated left ventricular stroke volume (SV) [5]. This underestimation was greater with increasing AS severity and was postulated to be due to intravoxel dephasing [6-17]. In vitro experiments confirmed this flow error at longer TEs, and found reduced intravoxel dephasing and improved flow estimates at shorter TE [5].

Shortening TE reduces the inherent higher order motion encoding [15], decreases the time available for the detrimental mixing of fast and slow moving spins, reduces the risk of turbulent velocity fluctuations disrupting the expected phase shift and thus improves the measured velocity's reliability and accuracy [5]. In a severely stenotic jet all of these effects are enhanced.

The aims of the study were to systematically study the reliability of PC aortic flow, peak velocity VTI and AVA estimates by CMR in patients with moderate or severe AS, and to investigate the *in vivo *effect, on these parameters, of reducing TE. We hypothesized that reducing the TE would improve the agreement between CMR echocardiographic estimates of AVA.

## Methods

### Study population

15 patients with isolated moderate or severe aortic stenosis (peak aortic velocity ≥3 m/s) were studied.

Patients were excluded if they had left ventricular impairment (ejection fraction <50%), atrial fibrillation, more than trace mitral or aortic regurgitation or other significant valvular disease, congenital heart disease, poor echocardiographic images, inability to undergo a CMR scan, or contraindications to CMR.

### Echocardiography

Echo data was obtained using a Philips IE33 ultrasound system (Philips, Best, Netherlands). All patients underwent a comprehensive 2D and Doppler echo within 1 hour of the CMR scan. Meticulous attention was paid to obtain the optimal velocity envelope and true peak trans-AoV velocity by sampling using continuous wave Doppler from multiple imaging windows (apical, right parasternal and suprasternal). Left ventricular outflow tract (LVOT) velocity profile was obtained by careful placement of the pulsed wave Doppler sample volume in the LVOT immediately below the AoV in an apical 5-chamber view. The LVOT diameter was measured by imaging the LVOT using the parasternal long axis view. LVOT area was estimated by assuming a circular orifice.

Analysis was performed off-line by a single experienced echocardiographer (AJK) blinded to the patients' CMR findings. Measurements were made according to American Society of Echocardiography guidelines [18] and averaged from 3 to 5 cycles. The velocity time intervals for the LVOT (VTI_LVOT_) and trans-AoV (VTI_AoV_) flow were obtained. The peak AoV velocity and VTI were reported from the window yielding the highest velocity signal. Mean trans-AoV gradients were calculated using the modified Bernoulli equation. Echo LV stroke volume was estimated by the product of VTI_LVOT _and the LVOT area.

### Magnetic Resonance Imaging

All MR data were collected on a Siemens 1.5 Tesla Avanto MRI scanner (Siemens Medical, Erlangen, Germany).

For CMR SV measurement the LV was imaged from apex to base with six equally spaced short axis slices and three orthogonal long axis slices orientated at 60° increments around the LV long axis. LV cines were obtained with a retrospectively gated steady state free precession (SSFP) sequence using a phased array surface coil and ECG triggering. Typical image parameters can be found in Table 1. Patients were in the supine position and all cines were acquired during a breath-hold ~15s in duration.

**Table 1:** Typical image parameters for each experiment

		SSFP cines	Phase Contrast variants		
			
			TE 2.79 ms	TE 2.00 ms	TE 1.50 ms
TR	(ms)	3.01	12.7	9.8	6.7
TE	(ms)	1.27	2.79	2.0	1.5
Flip angle	(degrees)	60	30	30	20
FOV	(mm)	320-360	320-360	320-360	320-360
Slice Thickness	(mm)	6	6	5.5	5.5
Acquisition Matrix		256 × 208	256 × 88	192 × 92	192 × 92
Lines acquired/Phase		11	4	4	7

Averages		1	1	1	2

Acquired Slices		3 long axis, 6-8 short axis	MPA, LVOT, 0 cm, 1 cm, 2.5 cm	0 cm, 1 cm, 2.5 cm,	0 cm, 1 cm, 2.5 cm

Typical VENC	(cm/s)	-	500	500	500

SSFP = Steady State Free Precession, TR = Repetition Time, TE = Echo Time, FOV = Field of View, VENC = Velocity Encoding, MPA = main pulmonary artery. LVOT = Left ventricular outflow tract, 0 cm/1 cm/2.5 cm = distance distal from aortic valve.

For quantitative flow measurements, PC images were obtained using the commercially released retrospectively gated breath-hold through plane velocity-encoding/velocity compensation technique with velocity compensation on the readout direction. Breath-hold acquisitions took ≈20 s, and calculated 25 phases. The standard TE was 2.79 ms. This sequence was modified to enable a TE of 2.0 ms by using the improved performance of current gradient hardware (45 and 200 T/m/s), halving the duration of the RF excitation pulse, and increasing the readout bandwidth from 390 Hz to 490 Hz. The TE was further reduced to 1.5 ms by maximising the gradient hardware and readout bandwidth (1530 Hz) and acquiring two averages to compensate for the reduced SNR. The in-plane resolution of the modified sequences was also adjusted (reduced base matrix size and increased phase resolution) to make the voxels more isotropic whilst maintaining a similar volume. Typical image parameters can be found in Table 1.

Through-plane flow was obtained in the following order: AoV leaflet tips (AoV_0 cm_), 1 cm beyond the AoV tips (AoV_1 cm_) in the aortic root, 2.5 cm beyond the AoV tips (AoV_2.5 cm_) just beyond the sino-tubular junction, LVOT, and main pulmonary artery (MPA). Both MPA and LVOT/AoV images were planned from paired orthogonal long axis SSFP cine images through the MPA and AoV respectively.

Aortic valve cine and flow imaging method: A standardised approach was followed to obtain short axis cine imaging for i) aortic valve planimetry, and, ii) to plan the optimal short-axis slice position for flow measurement (AoV_0 cm_). i). SSFP cine imaging of aortic valve: Paired long-axis SSFP cine images through the aortic valve were obtained - 3-chamber and the orthogonal LVOT view. The operator positioned the initial cine slice (6 mm slice thickness) at the AoV at end-diastole using the paired long axis cines. Our experience is that in moderate or severe AS when the valve opens in systole and moves down towards the apex it descends through this slice, which is therefore a useful initial position for obtaining cine images for planimetry of the limiting valve orifice. When images did not appear optimal the slice was repeated ± 6 mm. ii). Aortic valve phase contrast flow (AoV_0 cm_): The optimal short-axis slice position for measurement of peak trans-valvular velocities is at the vena contracta just at or beyond the anatomic valve orifice in systole. In straight pipes with planar circular orifice plates the vena contracta occurs about one orifice diameter downstream from the orifice [19]. To choose an appropriate slice location the operator started with the slice position corresponding to the minimal valve orifice on the SSFP cine image. Appropriate positioning was confirmed by checking in the paired long axis cines that the slice transected the proximal AS jet in mid systole immediately above the aortic valve leaflets. If necessary the slice position was adjusted to achieve this. This position was taken as AoV_0 cm_. A VENC scout sequence with VENC of 3.5 m/s, 4.0 m/s and 4.5 m/s was used to choose the appropriate VENC in the AoV_0 cm_, AoV_1 cm _and AoV_2.5 cm _acquisitions. When aliasing occurred at 4.5 m/s, flow was acquired at higher VENCs in 0.5 m/s steps.

### CMR analysis

The CMR SV was determined by interactively fitting a 3D LV finite element model to the images using the software package, CIMv4.6 (Auckland MRI Research Group, University of Auckland, New Zealand). This method has been previously validated in patients with cardiac disease, post mortem results from animal studies, and against PC velocity estimation of SV [20].

The PC data was analyzed by manually tracing around the LVOT, aortic root and MPA in each frame using ARGUS syngo MR 2004V (Siemens Medical Systems). The contours were exported to customised software, written in Matlab (Math-Works, South Natick, MA, USA), to apply the linear surface background phase correction process previously described by Lankhaar et al [21]. An estimate of the background phase was obtained by manually removing anatomically wrapped regions (due to spatial aliasing) and identifying stationary tissue using the suggested temporal velocity standard deviation threshold of 25%. Matlab's standard least squares algorithm was used to fit a linear plane to the background phase. Background phase correction was performed by subtracting the surface from the original velocity phase map.

The flow at each frame was calculated by multiplying the average velocity within each contour by its area. A PC estimate of SV (PC SV) was determined by summation of the net forward flow through the cardiac cycle.

Peak velocity for each frame was obtained as the maximum velocity of all pixels within the vessel. No neighbourhood averaging was used for peak velocity estimation, instead, as previously suggested by Nayler et al [22], the magnitude image's pixel value for the peak velocity must be above a certain threshold. Signal loss is an indicator of intravoxel dephasing and a loss of reliability in the PC estimate of velocity [5,22]. Normalised signal intensity (NSI) for each pixel was obtained by dividing their magnitude value with the average magnitude across the whole vessel obtained over the last 10 frames of diastole. (Diastolic blood, though not stationary, is the best representation of the minimum expected signal intensity when a pixel's velocity is reliable.) Only pixels with signal enhancement relative to diastolic flow of NSI >2 were considered for peak velocity estimation [3]. The velocity time integral was calculated by numerically integrating, using Simpson's rule, the area under the peak velocity versus time curve during systole.

The highest peak velocity (V_,pk_) was chosen to coincide with the highest phase contrast estimate of VTI (VTI_pk_) between AoV_0 cm _and AoV_1 cm_.

### Aortic valve area estimation using echo and CMR

For echo the AVA was estimated using the continuity equation as follows [23]:

Two CMR methods for estimating the AVA, both using the highest VTI estimate (PC VTI_pk_), were investigated.

1. AVA_vol _used the volumetric CMR SV estimate:

2. AVA_flow _used the phase contrast estimate of flow at the same AoV level as the highest VTI:

As an additional validation the CMR aortic valve area was also estimated by direct planimetry. The maximal visible aortic valve orifice in the short axis SSFP image obtained at the leaflet tips was manually traced as previously described. [24]

Severe AS was defined as AVA <1 cm^2^, or peak velocity >4 m/sec by echo [23].

### Statistics

Statistical analysis was performed with R for windows, v2.6.2 (The R foundation for statistical computing). Paired 2-tailed T-test was used to test differences between volumetric and PC estimates of SV and the effect of background phase correction. p < .05 was considered statistically significant.

Pearson linear regression (r^2^) was used to: a) compare the CMR SV with the PC SV at each level; b) to compare the peak velocity, VTI and AVA calculated using Doppler echo with CMR estimates. Agreement between methods was also assessed by Bland-Altman analysis (bias (mean of the differences between pairs of measures) and 95% limits of agreement (± 1.96SD of the difference between pairs of measures)).

## Results

Fifteen patients with moderate or severe AS were studied (11 men, age 71 ± 10.5 years). Ten patients had calcific aortic stenosis with trileaflet AoVs and five had bicuspid valves. By echo the mean aortic peak velocity was 4.2 m/s (range 3.3 m/s to 5.5 m/s), mean gradient 44.7 mmHg (range 25.6 mmHg to 75.2 mmHg) and AVA 0.85 cm^2 ^(range 0.52 cm^2 ^to 1.50 cm^2^). 12 patients (80%) had severe AS.

### Background phase correction data

The effect of the background phase correction on PC SV estimation is shown in Additional file 1. For TE 2.8 there was a statistically significant difference between the SV error before and after correction at the MPA and the LVOT; however, the mean SV errors were small (1.6% and 4.8% respectively) and no significant difference was observed at the AoV (AoV_0 cm_, AoV_1 cm _and AoV_2.5 cm_). The TE of 2.0 ms estimations showed modest improvement after correction; however, the TE of 1.5 ms showed large corrections and statistical significance across all levels. Therefore only the TE 1.5 ms data were phase corrected in subsequent analyses.

### CMR flow data

**Figure 1:**
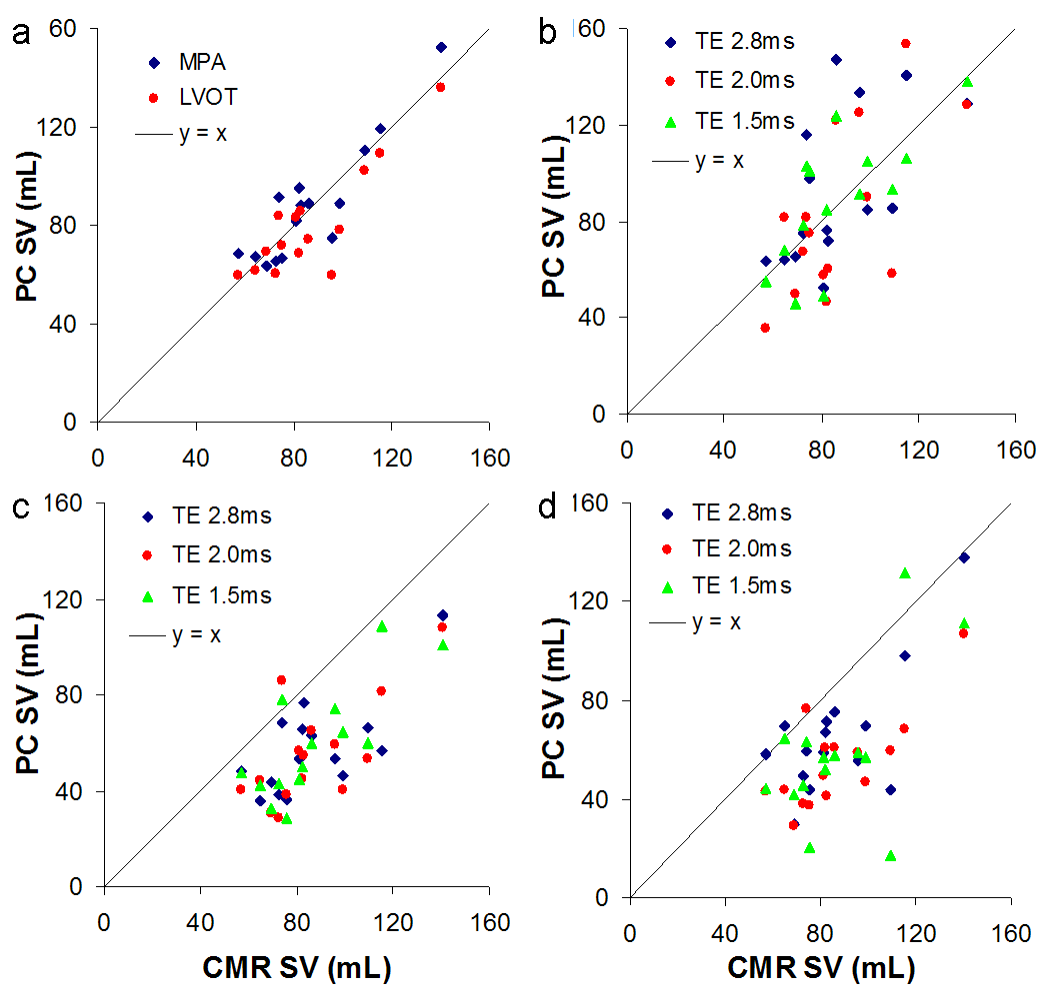
**PC SV at serial cardiac locations - MPA/LVOT (a), AoV_0 _(b), AoV_1 _(c) and AoV_2.5 _(d) versus CMR SV**. For the AoV_0 _to AoV_2.5 _levels the flow obtained using TEs of 1.5 ms, 2.0 ms and 2.8 ms are shown.

The mean CMR SV was 87.0 ± 21.8 mL. Table 2 and Figure 1 show the mean flow estimates and errors in PC estimation of flow at MPA, LVOT, AoV_0_, AoV_1 _and AoV_2.5_. In the absence of mitral regurgitation or left-to-right shunts we expect the flow at each level to be close to the CMR SV estimate. At a TE of 2.8 ms, the mean SV by phase contrast was very similar to CMR SV at the MPA and LVOT were very well correlated with minimal bias and narrow 95% limits of agreement, Table 2. Bias was small at AoV_0 cm_, but PC estimates significantly underestimated CMR SV at both AoV_1 cm _and AoV_2.5 cm_. This underestimation of flow beyond the AoV occurred across the range of TEs and was not improved by a TE as low as 1.5 ms.

**Table 2:** Comparison of the mean CMR SV with that obtained by PC in the MPA, LVOT and at serial AoV levels for various TEs.

	TE (ms)	Mean SV ± SD (mL)	Paired T-test p-value †	Bias ± 1.96SD (mL) *	Linear regression r^2^/p-value
CMR SV		87.0 ± 21.8			

MPA	2.8	88.3 ± 24.3	0.62	1.3 ± 20.2	0.82/< 0.01

LVOT	2.8	80.2 ± 21.5	0.03	-6.8 ± 21.9	0.75/< 0.01

AoV_0 cm_	2.8	93.5 ± 31.6	0.34	6.5 ± 50.7	0.34/< 0.01

	2.0	82.1 ± 35.0	0.47	-4.8 ± 51.4	0.44/< 0.01

	1.5	88.8 ± 27.2	0.78	1.5 ± 37.9	0.51/< 0.01

AoV _1 cm_	2.8	57.7 ± 19.9	< 0.01	-29.3 ± 31.2	0.50/< 0.01

	2.0	55.5 ± 22.1	< 0.01	-31.5 ± 33.2	0.49/< 0.01

	1.5	59.7 ± 23.8	< 0.01	-27.6 ± 29.9	0.62/< 0.01

AoV_2.5 cm_	2.8	65.8 ± 25.6	< 0.01	-21.1 ± 35.7	0.51/< 0.01

	2.0	54.6 ± 19.3	< 0.01	-32.3 ± 28.4	0.57/< 0.01

	1.5	58.6 ± 30.5	< 0.01	-28.6 ± 49.9	0.33/0.03

† Paired T-test with Bonferroni correction, comparison of PC SV at each level with the CMR SV

* Bias and the 95% limits of agreement determined by Bland-Altman analysis

CMR SV = left ventricular stroke volume by cardiac magnetic resonance, MPA = main pulmonary artery, LVOT = left ventricular outflow tract, AoV = aortic valve

The Bland-Altman bias ± 1.96SD, the paired T-test and the linear regression statistics between CMR SV and PC SV estimates are shown.

**Figure 2:**
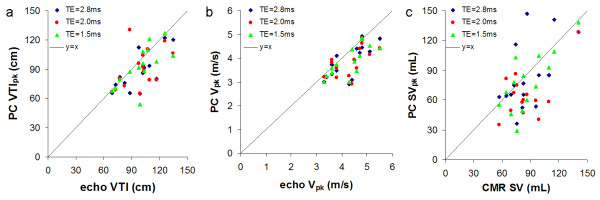
**Comparison of the echo Doppler peak velocity and echo Doppler VTI with the VTI_PC, max _(a), V_PC, pk _(b) and PC SV determined from the location of VTI_PC, max _in the aorta with CMR SV (c) for three TEs, TE = 2.8 ms, TE = 2.0 ms, TE = 1.5 ms**.

Although the mean flow at AoV_0 cm _was similar to the mean CMR SV, there was much poorer individual agreement at the AoV_0 cm _compared with the MPA (Table 2 and Figure 2). The Bland-Altman analysis 95% limits of agreement between CMR SV and PC SV in the MPA were half the agreement at the AoV_0 cm _at TE 2.8. Shortening the TE to 1.5 ms reduces the agreement and improves the correlation (Figure 1b).

At the max VTI_pk _level, the Bland-Altman analysis between CMR SV and PC SV exhibits a similar bias across the TEs, but the 95% limits of agreement and linear regression are improved at a the shorter TE of 1.5 ms, Table 3.

**Table 3:** Mean AVA, SVs, peak trans-AoV velocities and VTIs estimated by CMR and Doppler methods.

	SV (mL)	V_pk _(m/s)	VTI_pk _(cm)	AVA (cm^2^)	
	Mean ± SD (p-value)†	Mean ± SD. (p-value)†	Mean ± SD (p-value)†	Mean ± SD. (p-value)	
CMR	87.0 ± 21.8	-	-	-	

Echo	81.8 ± 17.5 (0.33)	4.28 ± 0.66	99 ± 19	0.85 ± 0.24	

PC SV at VTI_pk _level				AVA_vol_	AVA_flow_
TE = 2.8 ms	83.7 ± 33.9 (0.83)	3.87 ± 0.66 (< 0.01)	89 ± 20 (0.02)	1.00 ± 0.26 (0.07)	0.96 ± 0.43 (0.31)
TE = 2.0 ms	71.0 ± 32.0 (0.26)	3.81 ± 0.59 (< 0.01)	91 ± 20 (0.12)	0.97 ± 0.20 (0.05)	0.77 ± 0.21 (0.32)
TE = 1.5 ms	78.0 ± 29.6 (0.44)	3.89 ± 0.63 (< 0.01)	93 ± 21 (0.07)	0.95 ± 0.22 (0.10)	0.85 ± 0.30 (0.93)

† p-value <.05 for comparison in each column with Doppler data (V_pk_, VTI_pk _and AVA) or CMR SV (SV)

The Doppler AVA is derived using the continuity equation. PC peak velocity, and PC SV data are from the same AoV level that the PC VTI_pk _was obtained.

### CMR vs echo for peak velocity, velocity-time integral, SV, and AVA

#### Left ventricular SV by echo and CMR (Table 3, 4)

**Table 4:** Bland-Altman and linear regression analysis between echo and CMR methods for AVA estimation.

TE (ms)	SV (mL)	V_pk_ (m/s)	VTI_pk_ (cm)	AVA_vol_ (cm^2^)	AVA_flow_ (cm^2^)
	Bland-Altman bias ± 1.96SD				
Echo	-5.14 ± 38.6	-	-	-	-
2.8	1.83 ± 63.9	-0.41 ± 0.95	-10 ± 28	0.15 ± 0.59	0.11 ± 0.81
2.0	-10.9 ± 69.8	-0.39 ± 0.97	-9 ± 39	0.12 ± 0.44	-0.08 ± 0.63
1.5	-4.52 ± 41.8	-0.38 ± 0.95	-7 ± 30	0.11 ± 0.44	0.00 ± 0.39

	Pearson linear regression r^2^/p-value				

Echo	0.27/0.05	-	-	-	-
2.8	0.30/0.03	0.54/< 0.01	0.53/< 0.01	0.10/0.24	0.11/0.23
2.0	0.37/0.02	0.41/< 0.01	0.23/0.07	0.26/0.05	0.01/0.92
1.5	0.57/< 0.01	0.56/< 0.01	0.49/< 0.01	0.31/0.04	0.57/< 0.01

PC estimates of V_pk_, VTI_pk _and AVA were compared to echo data and SV estimates were compared against CMR SV.

The mean CMR SV and Doppler echo SV were similar (87 mL and 81.8 mL, respectively) but with relatively poor agreement at individual patient level, Table 4

#### Peak velocity and VTI data (Table 3, 4 Figures 2, 3)

**Figure 3:**
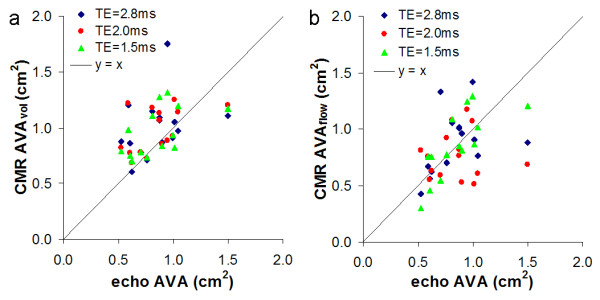
**Comparison of echo and CMR estimates of the AVA using the volumetric (a) and flow method (b) with three different TEs, TE = 2.8 ms TE = 2.0 ms TE = 1.5 ms**.

The peak velocity and VTI_PC _estimates generally occurred at the same location and they were predominantly found at the AV_0 cm _level (see Additional file 2).

CMR systematically underestimated both peak velocity and VTI compared with Doppler echo (Table 3). Except for this underestimation by CMR there was moderate agreement between the two methods (Fig 3, Table 4). Shortening TE showed no significant improvement in agreement by Bland-Altman or linear regression.

**Figure 4:**
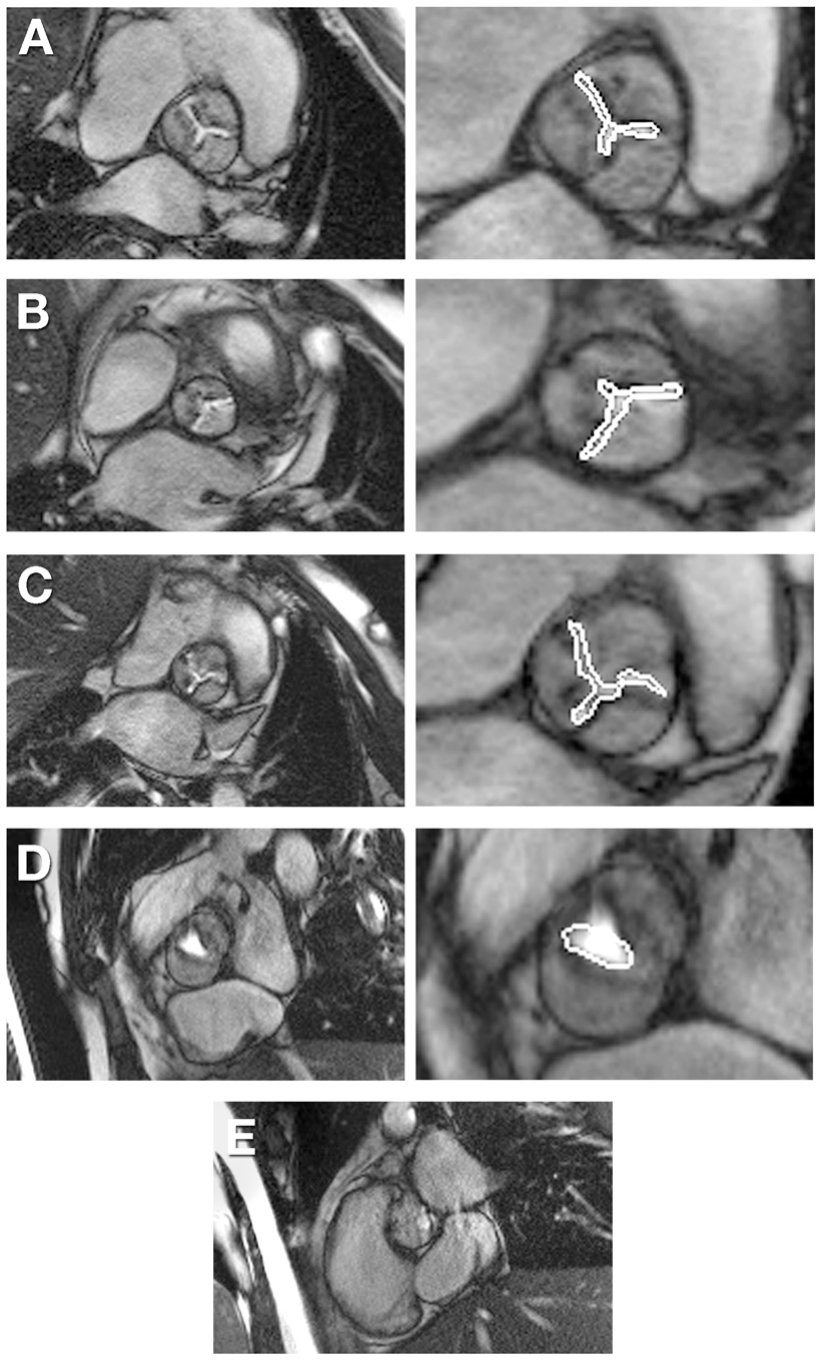
**The range of image quality in cine images obtained for AVA planimetry, and the corresponding planimetry are illustrated**. These range from good quality (A), to cases where images were sub-optimal due to poorly defined borders (B, C) or in-plane flow artefacts (D), to one case where planimetry was not considered possible (E).

#### AVA (Tables 3 and 4, Figure 4)

Two CMR methods of estimating AVA are compared with echo AVA. The agreement on Bland-Altman analysis is relatively poor between echo and both CMR methods with the 95% limits of agreement between the methods of at least 0.39 cm^2. ^Compared with echo, the CMR AVA_vol _method systematically overestimated echo AVA. The AVA_flow _method showed better agreement with echo at the shorter TEs. However at all TEs examined, both components of the calculation (PC SV and PC VTI_pk_) were systematically underestimated compared with echo data.

The CMR method which agreed best with echo AVA was direct planimetry of the valve from SSFP images. The Bland-Altman bias was -0.03 cm^2 ^with 95% limits of agreement ± 0.24 cm^2 ^in the 14 of 15 patients with technically adequate images.

## Discussion

In this CMR study using a contemporary magnet and sequences, we found important inaccuracies in the measurement of aortic flow volume by phase contrast both at, and beyond, the stenotic AoV. The PC SV had poor individual agreement with the CMR SV at the aortic valve level and the flow became underestimated beyond the aortic valve. The 95% limits of agreement were reduced when the TE was shortened to 1.5 ms; but agreement between echo and CMR AVA remained sub-optimal. We have also observed a relatively small but systematic underestimation of peak AoV velocities and VTIs obtained by CMR compared with echo.

### Stroke volume flow measurement in aortic stenosis

The comparisons of the gold standard CMR SV with the flows measured by PC at AoV level were highly variable, and those measured beyond the stenotic AoV were underestimated by between 20 to 30%. At the AoV level and at the level corresponding to the PC VTI_pk_, the 95% limits of agreement reduced with shorter TEs; however, although the correlations improved with shorter TEs, they were at least twice that observed in the MPA.

Prior to the jet's vena contracta the flow experience large spatial accelerations and is converging; after the vena contracta the flow begins to diverge and decelerate [25]. Accelerations have a stabilising effect on turbulence, thereby the turbulence's intensity is greatest distal from the vena contracta [10,25-27]. Shortening the TE, therefore reducing the higher order motion encoding, reduces the intravoxel dephasing and most probably explains the improved results close to the AoV with a TE 1.5 ms. At AoV_1 cm _and AoV_2.5 cm_, the presence of turbulence with a greater intensity ensures significant intravoxel dephasing errors were still present in-vivo even with the TE of 1.5 ms.

If possible, further reduction in the TE would be beneficial in decreasing the intravoxel dephasing and therefore improving the standard deviations of the flow measurements in the aorta towards those observed in regions relatively free of intravoxel dephasing such as the MPA. Shorter TEs should also help to remove the underestimation of PC SV distal to the AoV.

### Comparison between echo and CMR for peak velocity and VTI

Peak trans-AoV velocities and VTI_PC _underestimated that observed by Doppler echo by about 10% on average. CMR PC peak velocity and PC VTI_pk _may always underestimate Doppler echo, due to spatial and temporal averaging. More specifically, beat-to-beat variations of the stenotic jet's orientation within the desired slice may violate PC's assumption of identical flow patterns. In comparison, Doppler echo is an instantaneous measurement whose signal intensity (therefore the planimetry of the Doppler echo's velocity envelope) is dependent on the number of red blood cells moving at the same velocity [28].

### Assessment of AVA by CMR compared with echo

The CMR AVA_vol _overestimates echo AVA because the VTI_PC _is underestimated by CMR. The CMR AVAs_flow _are better calibrated to echo but only because both the PC SV and VTIs are proportionately underestimated. Decreasing the TE to 1.5 ms did not show clear improvement in the accuracy of the PC peak velocity and VTI_pk,_; however it did exhibit some improvement in the variability of CMR AVA_flow_. This was likely due to the improved variability of the PC SV estimate.

In contrast to our study, Caruthers et al [3] and Yap et al [4] obtained a better correlation between echo and PC AVA. This is in part because they included patients with mild AS.

Yap et al [4], in patients with aortic stenosis secondary to a bicuspid AoV, report a good correspondence between CMR SV measured by volumetric analysis and phase contrast (TE of 2.9 ms). But, their 95% limits of agreement increased from 0.1 ± 23.1 mL prior to the AoV to 1.5 ± 31.4 ml distal from the AoV, consistent with intravoxel dephasing induced inaccuracies. Several patients with moderate to severe AS showed marked variation between echo and PC methods and the volumetric (-0.01 ± 0.37) and flow CMR (0.02 ± 0.45) AVA methods had similar 95% limits of agreement to those reported in this study.

Caruthers et al [3] (TE of 3.1 ms) do not report flow or CMR SV comparisons. Only five patients had Doppler echo VTI estimates in excess of 0.8 m, all whom were underestimated with PC. The 95% limits of agreement (± ≈0.4 cm^2^) in their study are similar to those of the current study.

It is also possible that the type of valve lesion is important. In the current study 10 of 15 patients had calcific AS which may cause more turbulent flow than in a bicuspid valve. Subtle differences in slice location may also be important. Yap et al [4] placed their slices at the AoV leaflet at end-systole which is further towards the apex than in our study where it was placed at the AoV tips in mid-systole. In straight pipes with planar circular orifice plates the vena contracta occurs about one orifice diameter downstream from the orifice making the optimal position likely to be at or just beyond the aortic valve leaflet tips during early to mid-systole. In practice this is difficult to achieve precisely due to valve plane movement during the cardiac cycle and breath-hold variation. We used long axis cine imaging to plan short axis planimetry of the aortic valve and then optimised the slice position for flow at the aortic leaflet tips using both short axis and long axis cine images. If only flow information, without additional planimetry is required, a simplified method using just the long axis cines and transecting the visible jet in early to mid systole would probably lead to similar results.

Caruthers et al in a study using free breathing PC found subtle differences in slice position made no difference but it is possible that with the more accurate slice positioning with breath-hold sequences that position is important. Slice positioning either just upstream or too far downstream from the AoV would lead to underestimation of peak velocities and VTI and corresponding overestimation of AVA. We compared results from the AoV_0 cm _and AoV_1 cm _slice position to test whether a significant difference occurs due to accurate positioning, and found that although 70% of the peak velocities were found in the AoV_0 cm _slice, the peak velocities and VTIs were very similar, Table A2, suggesting that inter-acquisition variation due to factors such as breath-hold variation may be more important than small changes in position. In this study we did not have an intermediate slice half way between AoV_0 cmand _AoV_1 cm_. Further investigation is needed to understand whether such small differences in slice location are important, if they do it would potentially limit the generalisability of the technique.

### Clinical implications

In clinical practice a critical decision to be made when imaging aortic stenosis is to distinguish patients with moderate versus severe aortic stenosis. Before CMR PC AVA estimates are used clinically, improved techniques to measure PC SV are required. Despite erroneous flow data it is possible to obtain peak velocities, VTIs and/or AVAs which correlate moderately well with Doppler echo. However in this study the PC method does seem to systematically underestimate peak velocity and VTI by approximately 10% compared with Doppler echo. Although this is a relatively small calibration error it could lead to a clinically important misclassification of some patients with severe AS as having moderate AS if only the peak velocity is used to make this decision. Further study is needed to better understand the relationship between PC CMR and Doppler echo derived velocity data. In this study the best agreement with echo AVA was obtained by direct planimetry of the AVA from SSFP images. Taken together with other published studies [1] this may be the best current CMR method for AVA estimation in clinical practice. Despite good results this technique is critically dependent on accurate tracing of the slit-like orifice in bicuspid aortic valves and the more complex "Mercedes sign" shape of stenotic tricuspid valve orifice. The boundary definition can be poor due to partial voluming effects, signal voids due to severe calcification and turbulence, and development of a robust flow based method is desirable (Figure 4). Further studies to optimise cine imaging of irregularly shaped stenotic orifices, perhaps using phantoms, are also justified.

### Limitations

The larger gradients used to reduce TE to 1.5 ms exacerbate background phase error [7]. Recent studies have reported improvement after phase correction by either imaging a stationary phantom after the patient [29] or applying surface fits to stationary tissue [21]. The surface approach applied in this study showed significant improvement in the SV data only at TE 1.5 ms (see Additional file 1).

The best approach for background phase correction has yet to be determined, the imaging of a phantom is time consuming and surface fits may be susceptible to stationary tissue identification. The requirement to perform background phase correction may also be scanner and sequence dependent. Thus the findings presented in this paper are representative of a Siemens Avanto scanner only.

Estimation of peak velocity and VTI_PC _estimates by CMR are likely to be less susceptible to intra-voxel dephasing as accurate information in each pixel is not required. Even one pixel per phase with good signal intensity in the core of the jet could theoretically provide an accurate estimate; however, aberrant low signal intensity pixels should be excluded to prevent inaccurate peak velocity estimates [22]. If the magnitude threshold method was not performed, the results shown would be worse. Unfortunately for flow measurements, data from every pixel is required. The inclusion of voxels with low signal makes the flow estimate more prone to intra-voxel dephasing errors. Shortening the TE reduces the intra-voxel dephasing, improving the signal and therefore increasing the reliability of the PC SV estimate.

On most clinical systems, a magnitude thresholding technique and the velocity encoded magnitude images, where the signal loss may be more apparent, are not available. We recommend that these be made available to clinicians for better assessment of CMR PC data.

The lack of a true gold standard makes comparisons between Doppler echo and PC CMR measurements difficult. However we did find that echo AVA corresponded reasonably well with AVA obtained by manual planimetry of the anatomic valve orifice. This finding is similar to that reported in prior studies comparing echo and CMR planimetry suggesting that the echo is a reasonable external gold standard. An important consideration in using Doppler echo as a gold standard comparison is that for accurate estimation the Doppler echo beam needs to be well aligned with the jet direction or peak velocity and the VTI will be underestimated. Because of inter-subject variability in aortic stenotic jet direction in relation to standard echo windows, accurate estimation is dependent on an experienced sonographer systematically sampling multiple echo windows (as was done in this study).

Accuracy of echo assessment of AVA is limited by difficulties in accurately measuring the LVOT area which is required for Doppler echo's estimation of AVA. A hybrid approach [30] utilising the most reliable data from echo and CMR may provide the most robust assessment of AVA until PC methods improve. The strength of echo is in instantaneous measurement of peak velocities and the VTI using Doppler. CMR can provide the most reliable estimate of SV in the absence of mitral regurgitation or by phase contrast flow estimation in the LVOT or MPA (in the absence of aortic regurgitation). AVA estimation using a CMR SV estimate and the Doppler echo VTI is likely to be the most reliable current non-invasive flow-based method.

## Conclusion

PC derived SV measured in the stenotic jet was unreliable, underestimated and not satisfactorily corrected by reduction in echo time to 1.5 ms in this study. Despite this the AVA by CMR correlated better with echo with a reduction in TE to 1.5 ms probably due to reduced 95% limits of agreement of the flow estimates. However, for estimation of AVA in more severe AS, improved techniques to measure this flow are required. Furthermore, the PC method does seem to systematically underestimate velocity and VTI data by approximately 10% compared with Doppler. Further study is needed to better understand the relationship between CMR and Doppler derived velocity data.

## Competing interests

The authors declare that they have no competing interests.

## Authors' contributions

KOB participated in the design of the study and analysis procedure, performed statistical and Background phase analysis, interpreted results and drafted the manuscript. RG coordinated and designed the study, supervised the data acquisition and performed the analysis of the MR images. AG modified the PC sequences to achieve shorter TEs and provided technical advice on the design of the protocols. BC helped to design the study, the analysis procedure and in the interpretation of the results. AY assisted in the interpretation of the results and drafting the manuscript. AK conceived the study, analysed the Echo data, participated in the data acquisition, interpreted the results and drafted the manuscript. All authors read and approved the final manuscript.

## Supplementary Material

Additional file 1 SV before and after phase correction. Click here for file

Additional file 2 Comparison of the VTI and peak velocity at the AoV_0 cm _and AoV_1 cm _levels. Click here for file
