# Supplementary material for: Aortic valve stenotic area calculation from phase contrast cardiovascular magnetic resonance: the importance of short echo time
Source: J Cardiovasc Magn Reson. 2009 Nov 19;11(1):49. doi: 10.1186/1532-429X-11-49 (PMC2785795; doi:10.1186/1532-429X-11-49)
Supplement: Additional file 1 — SV before and after phase correction. [file 1532-429X-11-49-S1.DOC]

| Position | Sequence | Background Phase correction | | | | |
| --- | --- | --- | --- | --- | --- | --- |
|  |  | Mean SV error ± SD (%) | | Paired  T-test  p-value | Pearson linear regression  r^2^ / p-value | |
|  |  | Before | After |  | Before | After |
| MPA | TE=2.8ms | 1.9 ± 12.5 | -5.8 ± 12.4 | <0.01 | 0.82 / <0.01 | 0.82 / <0.01 |
| LVOT | TE=2.8ms | -7.2 ± 12.5 | -10.2 ± 12.9 | 0.01 | 0.75 / <0.01 | 0.76 / <0.01 |
| AoV_0cm_ | TE=2.8ms | 8.5 ± 29.9 | 10.5 ± 28.4 | 0.51 | 0.34 / <0.01 | 0.38 / <0.01 |
|  | TE=2.0ms | -6.4 ± 29.2 | 1.4 ± 26.6 | 0.56 | 0.44 / <0.01 | 0.41 / <0.01 |
|  | TE=1.5ms | -21.7 ± 55.6 | 6.0 ± 26.91 | 0.07 | 0.14 / 0.18 | 0.51 / <0.01 |
| AoV_1cm_ | TE=2.8ms | -33.3 ± 16.0 | -32.3 ± 18.2 | 0.69 | 0.50 / <0.01 | 0.57 / <0.01 |
|  | TE=2.0ms | -36.3 ± 19.3 | -25.2 ± 24.1 | <0.01 | 0.49 / <0.01 | 0.45 / <0.01 |
|  | TE=1.5ms | -63.5 ± 40.8 | -20.8 ± 13.5 | <0.01 | 0.16 / <0.01 | 0.62 / <0.01 |
| AoV_2.5cm_ | TE=2.8ms | -24.1 ± 20.1 | -19.4 ± 18.5 | 0.14 | 0.51 / <0.01 | 0.66 / <0.01 |
|  | TE=2.0ms | -37.1 ± 15.5 | -22.8 ± 17.4 | <0.01 | 0.57 / <0.01 | 0.57 / <0.01 |
|  | TE=1.5ms | -76.1 ± 50.6 | -20.0 ± 14.7 | <0.01 | 0.49 / <0.01 | 0.33 / 0.03 |

The mean SV error was obtained by comparing it to the CMR SV, SV error = (PC SV – CMR SV)/CMR SV.
