# Supplementary material for: Aortic valve stenotic area calculation from phase contrast cardiovascular magnetic resonance: the importance of short echo time
Source: J Cardiovasc Magn Reson. 2009 Nov 19;11(1):49. doi: 10.1186/1532-429X-11-49 (PMC2785795; doi:10.1186/1532-429X-11-49)
Supplement: Additional file 2 — Comparison of the VTI and peak velocity at the AoV0 cm and AoV1 cm levels. [file 1532-429X-11-49-S2.DOC]

|  |  | | TE=2.8ms | TE=2.0ms | TE=1.5ms |
| --- | --- | --- | --- | --- | --- |
| Proportion of VTI_pk_ occuring at AoV_0cm_ | | | 73% | 73% | 71% |
| Proportion of VTI_pk_  and V_pk_ occuring at same location | | | 93% | 80% | 79% |
| Comparison between AoV_0cm_ and AoV_1cm_ | | |  |  |  |
| VTI (cm) | | Mean diff. ± SD | 14.1 ± 21.5 | 7.1 ± 19.3 | 5.2 ± 17.6 |
| peak V (m/s) | | Mean diff. ± SD | 0.28 ± 0.52 | 0.21 ± 0.37 | 0.22 ± 0.33 |
